# Opportunities and Challenges in the Care of Patients with Somatic Complaints and Patients with Additional Work-Related Anxiety—A Mixed Methods Study

**DOI:** 10.3390/ijerph23010125

**Published:** 2026-01-20

**Authors:** Lara Kleist, Franziska Weißenstein, Beate Muschalla, Lukas Kühn, Eileen Wengemuth, Kyung-Eun (Anna) Choi

**Affiliations:** 1 Center for Health Services Research, Brandenburg Medical School Theodor Fontane, Fehrbelliner Str. 38, 16816 Neuruppin, Germany; lara.kleist@mhb-fontane.de (L.K.); lukas.kuehn@ukmuenster.de (L.K.); eileen.wengemuth@mhb-fontane.de (E.W.); anna.choi@mhb-fontane.de (K.-E.C.); 2 Psychotherapy and Diagnostics, Technische Universität Braunschweig, 38106 Braunschweig, Germany; b.muschalla@tu-braunschweig.de; 3 Health Services Research, Research Center Medical Imaging and Artificial Intelligence (MIAAI), Danube Private University (DPU) GmbH, Steiner Landstraße 124, 3500 Krems-Stein, Austria

**Keywords:** work and health, employee health, rehabilitation, work anxiety, occupational health

## Abstract

**Highlights:**

**Public health relevance—How does this work relate to a public health issue?**

- Work-related anxiety can result in prolonged work incapacity and decrease the chances of returning to work, causing high economic cost.
- Despite available therapeutic approaches, little is known about patient-reported challenges and support needs across healthcare, workplace, and rehabilitation contexts.

**Public health significance—Why is this work of significance to public health?**

- Reveals substantial differences in barriers and support needs between patients with and without work-related anxiety in somatic healthcare settings.
- Highlights the need for preventive, multi-level approaches involving health professionals, employers, and organizational systems to address work-related anxiety before it progresses to prolonged work disability.

**Public health implications—What are the key implications or messages for practitioners, policymakers, and/or researchers?**

- Practitioners should implement work-related interventions specifically tailored to the needs of patients with work-related anxiety.
- Policymakers should promote mental health literacy training for both workers and supervisors to enable early identification and timely support for employees facing work-related mental health challenges.

**Abstract:**

Background: Work-related anxiety can result in prolonged work incapacity and reduce return-to-work probabilities. Despite the prevalence of work-related anxiety in somatic rehabilitation settings, there has been little research examining the experiences of affected patients from a public health perspective. This research project aims to address this gap by providing initial insights into the care provided to patients with somatic complaints and patients with additional work-related anxiety. Methods: A sequential mixed methods approach was employed, beginning with semi-structured interviews (2022, *n* = 18 orthopedic rehabilitation patients), followed by questionnaire distribution (2023, *n* = 53). Qualitative analysis distinguished between patients with higher (JA) and lower (nJA) Job Anxiety Scale scores (cut-off 2.5). Results: The findings highlight notable differences between JA and nJA patients. JA patients often report that they face unmet psychological needs, limited work-related treatment focus, financial barriers, and inadequate occupational support, relying more on self-initiative for reliable information. In contrast, nJA patients appear to benefit from stronger social networks, stable financial resources, and improved access to healthcare. Both groups report mixed experiences with workplace support. For professionals the findings underline that JA patients are specifically in need of work-related interventions, even patients themselves remind about this. Conclusions: The findings illustrate significant differences between JA and nJA patients in terms of their experiences, challenges, and support needs within healthcare, workplace, and rehabilitation contexts. While qualitatively insightful, these findings are pilot and explorative and warrant further research. Trial registration: DRKS00029004 (25 May 2022).

## 1. Introduction

Work has numerous positive effects, including providing income, promoting social engagement, and offering a sense of purpose. However, the work environment can also trigger anxiety for some individuals. Common triggers of workplace anxiety include performance expectations and evaluations, competitive conflicts, pressures from third parties, the risk of accidents, health hazards, unpredictable changes, and existential threats [1]. Work-related anxiety refers specifically to anxiety that arises in the context of the workplace and can be distinguished from other anxiety disorders both clinically and empirically [2]. According to Muschalla [3], individuals are considered to have a pathological condition if they experience significant impairment or distress in their daily work or personal life due to their work-related anxiety.

In Germany, work-related anxiety is primarily recognized in rehabilitation settings [3]. The rehabilitation system in Germany is an integral part of the welfare state, aimed at reintegrating individuals with health limitations into professional and social life. It encompasses medical and vocational rehabilitation measures. Medical rehabilitation focuses on stabilizing and enhancing the health of individuals affected by a condition. It is often carried out in specialized inpatient rehabilitation clinics and includes treatments such as physiotherapy, psychotherapy, and other medical therapies. Vocational rehabilitation is designed to reintegrate individuals into the workforce. Organizations such as the German Pension Insurance or the Federal Employment Agency provide financial support for measures like retraining, further education, or workplace adjustments. Various providers, including health insurance funds, pension insurance agencies, accident insurance institutions, and social assistance agencies finance the rehabilitation system. It is legally anchored in the Social Code (Sozialgesetzbuch, SGB) and operates under the principle of “rehabilitation before retirement,” aiming to keep as many people as possible active in the workforce.

Employers play a crucial role in vocational rehabilitation by adapting workplaces, supporting retraining, and collaborating with rehabilitation providers. They can offer flexible work arrangements, promote workplace health, and assist with reintegration after illness. By fostering inclusivity and removing barriers, employers help ensure the success of vocational rehabilitation efforts. In Germany, employers must support vocational rehabilitation under SGB IX by meeting a 5% disability employment quota, adapting workplaces, and protecting employees with disabilities from dismissal. They must collaborate with rehabilitation agencies, use available funding for adjustments, and take preventive actions to ensure workplace inclusion. Furthermore, they must offer a company reintegration management (BEM) after six weeks of employee illness within a year.

Among rehabilitation patients, those with orthopaedic conditions experience the highest levels of work-related anxiety and the most extended periods of disability, averaging 20.6 weeks over a year, compared to neurological and cardiac patients [4,5]. In somatic rehabilitation, 20–27% of patients report experiencing work-related anxiety. In the context of somatic complaints, factors at work can trigger this anxiety, which may also come along with physical symptoms over time. Conversely, somatic complaints can instill a fear of not being able to meet work demands, thereby triggering work-related anxiety. Additionally, individuals may fear that their physical issues will recur upon returning to work [1,6,7,8]. If work-related anxiety goes undiagnosed, it can result in prolonged work incapacity and decrease the chances of returning to work [9]. Consequently, work-related anxiety directly impacts participation in working life and contributes to some of the highest economic costs associated with mental health issues [10].

The existing literature outlines various therapeutic approaches for addressing work-related anxiety in rehabilitation, hospital, and outpatient settings [2,3]. Additionally, preventive recommendations and guidance are available for companies [9,10]. When treating work-related anxiety, it is crucial to consider not only the psychological aspects but also the actual work environment [1]. Muschalla & Linden [1] highlight the significance of collaboration among different care providers, including general practitioners, rehabilitation professionals, return-to-work partners, and employers, for effective treatment and prevention of work-related anxiety. However, previous research has primarily focused on the clinical context.

From a healthcare research perspective, it remains unclear what challenges and opportunities exist for patients with work-related anxiety and somatic complaints that might positively or negatively impact their recovery process. Therefore, this research project aims to provide preliminary insights into the care received by patients while investigating potential challenges and opportunities. In line with this goal, our research question is: *Which facilitating and hindering factors can be identified in the recovery process for patients with somatic complaints and patients with somatic complaints and additional work-related anxieties?*

## 2. Materials and Methods

### 2.1. Study Design

#### 2.1.1. Qualitative Methods

The project employs a dependent, sequential mixed methods approach, as outlined by Schoonenboom & Johnson [11]. Initially, semi-structured face-to-face interviews were conducted with patients at a Rehabilitation Clinic in Brandenburg, who were being treated for somatic complaints, from June to October 2022. The early interviews served to pilot the interview guide, and no significant adjustments were necessary. Participants also completed a short questionnaire covering sociodemographic information, brief medical history, and work-related anxiety (items on health-related anxieties of the Job Anxiety Scale, [12]). All interviews were audio-recorded, transcribed verbatim, and analysed using qualitative content analysis [13]. Participants were informed that their data would be handled confidentially, that they could interrupt or terminate the interviews at any time, and that they had the option to review their transcripts.

#### 2.1.2. Quantitative Methods

Building on the interviews, a questionnaire was developed and distributed to patients at the Rehabilitation Clinic and an orthopaedic department of a hospital in Brandenburg from May to September 2023. Patients were invited to participate in the survey either online or via paper-and-pencil format. Recruitment occurred on-site during information events at the Rehabilitation Clinic and through direct engagement by hospital staff at the orthopaedic clinic, supported by project flyers. The questionnaire includes both self-developed and existing questions on sociodemographic and disease history aspects, the healthcare system (e.g., stay in the rehabilitation clinic or hospital, health-literacy sensitive communication [14]), the individual situation (e.g., financial losses due to the illness, family situation), work situation, and work-related anxiety (items on health-related anxieties of the Job Anxiety Scale [12]). Due to the exploratory nature of the survey, no predetermined sample size was established; instead, the focus was on obtaining the highest possible number of questionnaire responses during the recruitment phase. The study is registered with the German Clinical Trial Register (DRKS00029004).

### 2.2. Study Participants

#### 2.2.1. Qualitative Study Participants

A total of 18 patients undergoing orthopedic rehabilitation were interviewed, with each interview lasting approximately 42 min (ranging from 19 to 81 min). As one participant did not fill out the short questionnaire, results of 17 interviews were included in data analysis. Of the participants, six were male and 11 were female, and 16 were employed. The average age of the participants was 52.71 years, with a standard deviation of 9.04. On average, the interviewees scored 1.95 (SD = 1.28) on the Job Anxiety Scale, where “0” indicates low and “4” indicates high work-related anxiety [15]. While seven patients achieved higher scores (≥2.5), ten achieved lower scores (<2.5) (1 missing).

#### 2.2.2. Quantitative Study Participants

Participants had to meet the following criteria: they were between 18 and 67 years old, had a diagnosis of (chronic) somatic illness, were currently employed or had worked in the past, and were fluent in German. Patients who had already participated in the interviews were not eligible to take part in the questionnaire survey.

A total of 53 questionnaires were collected as part of a quantitative survey. This included 35 patients from the orthopedic rehabilitation clinic and 18 from the orthopedic unit of an acute hospital. Also, questionnaires with missing data were included in analyses. Among the participants, 25 were male and 25 were female (three responses were missing). Out of these participants, 34 were working full-time, 14 were part-time, one was in marginal employment, and one was seeking work (three responses were missing). The average age of the participants was 55.64 years, with a standard deviation of 10.31. On average, the participants scored 1.50 (SD = 0.99) on the Job Anxiety Scale [15]. Nearly 30% of patients in the rehabilitation setting exhibited high scores (≥2.5) on the Job Anxiety Scale, compared to only about 6% in the hospital setting.

### 2.3. Data Analysis

#### 2.3.1. Qualitative Data Analysis

The interviews were analyzed using MAXQDA Analytics Pro 20 (Berlin, Germany). Initially, all interviews were divided among three researchers, who were rehabilitation scientists and psychologists. They independently and inductively defined possible domains. After discussions, they reached a consensus on the main domains. LKl (rehabilitation scientist) and FW (psychologist) then further elaborated on the sub-domains and made slight adjustments to the main domains through an iterative process. Once the coding system was finalized, LKl and FW each coded nine interviews according to the established main and sub-domains. To ensure accuracy, they cross-checked each other’s coding. As a result, three main categories were identified: individual situation, work, and health care.

**Figure 1:**
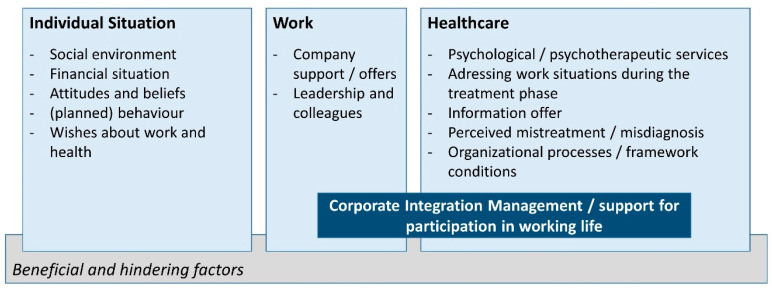
Coding System.

The main category *individual situation* includes all subcategories in which interview statements regarding personal living circumstances (*social environment* and *financial situation*), (*planned) behavior*, personal *attitudes and beliefs*, and *wishes about work and health* could be identified. The category *work* encompasses all statements providing insights into *company support*/*offers*, *leadership*, and *colleagues*. The main category *healthcare* includes statements indicating the use of and/or desire for *psychological or psychotherapeutic services*, addressing the extent to which work situations were considered in the treatment context, and how patients learned to cope with their health situation in (work) everyday life during treatment (*addressing work situations during the treatment phase*). Additionally, it includes statements on the availability of information (*information offers*), *perceived mistreatments*/*misdiagnosis*, and *organizational processes*/*framework conditions*. *Corporate integration management*/*support for participation in working life (LTA)* was defined as an overlapping subdomain of *work* and *healthcare.* Both facilitating and hindering factors were distinguished in all three main categories. The coding system is illustrated in Figure 1.

In presenting the qualitative results, a distinction is made between patients with higher scores on the JAS (≥2.5), indicating potentially existing work-related anxiety, and those with lower scores (<2.5). It should be noted that these cutoff scores represent a screening threshold and do not constitute a clinical diagnosis, which would require specialist evaluation. References to quotes found in Table S1, Supplementary Materials are consecutively numbered and marked with ‘JA’ for patients with higher values, and with ‘nJA’ for patients with lower values on the Job Anxiety Scale. Some statements from JA patients highlight existing fears or discomfort regarding work, e.g.,

*“And I/I just can’t anymore. I really have panic attacks when I have to call in and report sick. I really/I measured my pulse during the (laughs)/ I actually had a pulse of 140. My heart is racing (…) I can’t do it anymore. I/I can’t even sleep the night before.” (RH-13)*

*“I even took medication before I, uh—don’t laugh—before I was about to go to that meeting at work.” (RH-05)*

*“And as I said, I was so drained, I really spent the whole night in bed with trembling knees, kept waking up because it was just such an exhausting day—those smells, the heat, that sticky feeling.” (RH-06)*

*“You can hardly sleep anymore because (A) you’re in pain and then (B) you’re worried about how things will progress, how it will go on. So/ and you DO want to work. It’s not like I don’t want to work, although slowly, it’s starting to scare me. (laughs) Yeah.” (RH-16)*

However, it is important to note that these statements do not allow for a diagnosis of existing work-related anxiety.

#### 2.3.2. Quantitative Data Analysis

The questionnaire data were analyzed using descriptive analyses in IBM SPSS Statistics 23 (Armonk, NY, USA), including frequencies, means, and standard deviations.

## 3. Results

### 3.1. Integrated Qualitative and Quantitative Findings

The results of the qualitative interviews and the quantitative survey are presented together below. The structure follows the coding system; the quantitative data were included wherever they directly related to the qualitative data or could complement them with numerical insights. As group differences cannot be calculated due to sample size, the results of the quantitative survey are presented in a consolidated manner.

### 3.2. Individual Situation

#### 3.2.1. Social Environment

Patients of both groups reported receiving social support from friends and family. They discussed their health situation with friends or family (JA1) and experienced emotional support (nJA1). Practical support in the form of concrete actions helped patients in their daily lives (JA2) and, in some cases, ultimately led to a medical treatment taking place at all (JA3 & nJA2). Patients also reported that their social situation influenced their professional situations (nJA3 & JA4).

The interviews indicate that nJA patients are more likely to forgo treatment due to a perceived sense of obligation toward their family (nJA4), while JA patients report that their health or work challenges are negatively influenced by familial dynamics (JA5, JA6) and emphasize the need for greater support from family and friends (JA7). Furthermore, information from their social environment helped nJA patients find suitable doctors, and retraining opportunities or access services more quickly (nJA5). In contrast, negative experiences with medical treatments within their social environment led some JA patients to reject certain medical treatments (JA8).

*“It was supposed to be a fifth week, uh, but that would have been over Christmas, (…) and since I’m rarely at home, of course, I have to travel. Uh, I have six grandchildren, and I can’t keep putting them through it with Grandpa constantly being away.” (nJA4, RH-03)*

*“(…) since my [family member] was the boss in the (…) department there and he has a bit of a strange character (laughs), you know, a bit of a “know-it-all” and a bit of a control freak. (…) they asked, ‘Are you related to [Name]?’ I said, ‘Yes, I’m his [family member].’ That was it. They started ignoring me and… well.” (JA4, RH-06)*

In the quantitative survey, individuals indicated that they have not used health care services due to their family situation (e.g., childcare, caregiving for relatives) at least once (*N* = 28). This did not apply to 31 individuals.

#### 3.2.2. Financial Situation

The financial situation of nJA patients allowed access to health care services or necessary aids (nJA6). Additionally, it is relevant for them to what extent other people in their private environment are dependent on their financial situation, or not (nJA7). JA patients, on the other hand, were unable to access healthcare services or necessary aids due to their economic situation (JA9 & JA10). In the survey, *N* = 14 individuals reported that they had refrained from utilizing healthcare services at least once because they could not afford the financial losses (e.g., due to receiving sick pay). In comparison, this did not apply to 35 individuals.

#### 3.2.3. Attitudes and Beliefs

In the interviews, nJA patients reported that employers and the support system are largely unable to provide practical assistance with work-related challenges (nJA8, nJA9). In contrast, participants in the JA group were more inclined to believe that employers are unwilling to help (JA11, JA12). They generally expressed a more skeptical attitude toward employers (JA13, JA14) but still considered them responsible for providing support with regard to health-related matters (JA15). Additionally, JA patients approached rehabilitation with the belief that it would be ineffective (JA16).

*“Well, how is he [the employer] supposed to react? There are no options to react.” (nJA8, RH-03)*

*“If my HR manager knows I have back problems (laughs), I don’t think he really cares about that anyway.” (JA11, RH-10)*

In the survey, *N* = 26 individuals (somewhat) agreed with the statement, “My employer could support me in health-related matters if they wanted to,” while *N* = 10 individuals (somewhat) disagreed with it. Twelve individuals stated that they could not assess this. Furthermore, *N* = 33 individuals (somewhat) agreed with the statement, “My employer is responsible for my health,” while *N* = 13 individuals (somewhat) disagreed with it.

#### 3.2.4. (Planned) Behavior

Some patients report a desire to pursue retraining to change their professional situation (JA17 & nJA10). Some, however, exhibited more health-damaging behavior by continuing to work despite their complaints and rarely seeking medical attention (nJA11 & JA18). Furthermore, patients reported that while they would like to change their situation, they do not do so for various reasons, some of which are unknown to them (JA19, nJA12, nJA13). While interviewees from both groups addressed the desire for change and the accompanying resilience required to achieve it, nJA patients were more likely to report engaging in health-promoting behaviors, e.g., changing current job (position) (nJA14).

#### 3.2.5. Wishes About Work and Health

The statements categorized as wishes primarily address employers and managers. On the one hand, these wishes revolve around the desire for greater understanding and consideration for the person affected by the illness, and on the other hand, for creating working conditions that are more conducive to health (nJA15, JA20, JA21, nJA16, JA22). Furthermore, wishes were also expressed regarding the care situation, for example, for more individualized treatment (nJA17) and more information for those affected (JA23).

### 3.3. Work

#### 3.3.1. Company Support/Offers

Regarding company support and offers, interviewees from both groups reported both positive and negative experiences. They received support, for example, through the works council (nJA18), changes in work tasks (nJA19), the representative body for severely disabled employ-ees, the integration specialist service, or the staff council (JA24). Other patients, however, also reported a lack of support from their company. For example, measures were announced but never implemented (nJA20), employers refused to sign essential documents intended to provide support in the workplace (JA25) or were unwilling to offer affected individuals a different position within the company (JA26).

*“Due to the illness, it eventually got to the point where, as I said, I just couldn’t do it anymore. But they know that, so they’re already trying to take me out of some tasks a bit. I’d rather drive the car than do other things.” (nJA19, RH-09)*

*“(…) at [employer], we have a special workplace team, the representative body for severely disabled employees, the integration specialist service, and the staff council, and I am well supported there.” (JA24, RH-16)*

In the survey, *N* = 21 individuals stated that their employer (somewhat) supports them in adapting work tasks to their health needs, *N* = 16 individuals (somewhat) disagreed with this, and *N* = 5 individuals stated that they could not assess this.

#### 3.3.2. Leadership and Colleagues

Regarding their supervisors and colleagues, interviewees from both groups report both positive and negative experiences. Some supervisors were very understanding in dealing with the affected person (nJA21), provided support (nJA22), and focused on their strengths (JA27). Other supervisors, on the other hand, engaged in negative gossip (nJA23), showed no willingness to find a way to retain the employee in the workplace (JA28), and demonstrated a lack of appreciation for the employee as a person (nJA24). In addition, workflows were changed without any apparent reason for the patients (JA29), individual needs were not taken into consideration (JA30), or even dismissals were issued due to the illness (JA31). Regarding colleagues, interviewees experienced support (JA32), compassion (JA33), and solidarity. On the other hand, however, there were also reports of rivalry (JA34) and envy among colleagues (nJA25).

*“He [the supervisor] was very understanding, and uh, I had no issues.” (nJA21, RH-14)*

*“(…) but then I had a new boss, and she was very supportive. She asked, ‘What are your strengths?’” (JA27, RH-16)*

The survey assessed the extent to which participants feel they can confide in their supervisor regarding health matters, with *N* = 26 participants who (somewhat) agreed, and *N* = 23 participants who (somewhat) disagreed. On the other hand, it was assessed how much under-standing the supervisor shows for the participants’ health situation, with *N* = 28 participants who (somewhat) agreed, and *N* = 21 participants who (somewhat) disagreed. Regarding colleagues, the survey assessed to what extent participants feel they can confide in their colleagues regarding health matters, with *N* = 32 participants who (somewhat) agreed, and *N* = 17 participants who (somewhat) disagreed. On the other hand, it was assessed how much understanding their colleagues show for the participants’ health situation, with *N* = 31 participants who (somewhat) agreed, and *N* = 17 participants who (somewhat) disagreed.

### 3.4. Healthcare

#### 3.4.1. Psychological/Psychotherapeutic Services

While JA patients express a desire for psychological support (JA35), this desire remains partially unmet in the rehabilitation context (JA36). nJA patients report positive experiences when psychological support is provided, although a certain skepticism is sometimes evident in their statements (nJA26). The survey revealed that *N* = 31 individuals (somewhat) had sufficient opportunities to speak with psychologists if needed (*N* = 10 disagreed with this (somewhat)). Furthermore, 16 individuals expressed a desire to (continue) receiving psychological or psycho-therapeutic help, while *N* = 19 disagreed, and *N* = 14 individuals were unsure about this.

*“I told the doctor that I… I included everything in the application. Contact with a psychologist, yes. I think if you talk to someone and they give you advice, it might make a difference.” (JA35, RH-05)*

#### 3.4.2. Addressing Work Situations During the Treatment Phase

While nJA patients frequently reported that their current occupational situation was addressed within the care context, for example, in rehabilitation settings, from general practitioners, psychologists, or orthopedic specialists (nJA27 & nJA28, nJA29), JA patients indicated that their occupational situation was not (adequately) addressed, either during rehabilitation or by stakeholders outside the rehabilitation setting (JA37).

*“We talk about everything. He [the general practitioner] also asks about work processes and so on—he’s very knowledgeable. We try to change some things, (…)” (nJA28, RH-09)*

*I: “Okay. And to what extent do these doctors address your work situation?” P: “Not at all. They’re not interested. Not at all. Zero.” I: “Would you have wanted that?” P: “Uh, yes. (laughs) Yes. (…)” (JA37, RH-16)*

The survey revealed that the majority of patients agreed that professional aspects were adequately addressed within the care facility. However, some individuals (somewhat) disagreed with the related statements (see Table 1).

#### 3.4.3. Information Offer

Patients received information on health and work-related topics, such as the possibility of retraining, counseling and support services, or the general option of rehabilitation, from sources like general practitioners, informational events during rehabilitation, social counseling, self-help organizations, or other patients. However, especially JA patients reported to have taken the initiative themselves, particularly if they felt poorly advised (JA38) or if they generally felt that no reliable information was provided from any source (JA39).

#### 3.4.4. Perceived Mistreatment/Misdiagnosis

Patients of both groups occasionally reported experiences of perceived “misdiagnoses or mistreatment.” For example, one patient stated that his/her pain was attributed to depression, despite medical reports indicating physical causes for the pain (JA40). A different patient expressed feeling dismissed regarding their complaints (JA41). Another patient mentioned being discharged from the hospital with a very positive prognosis, despite feeling that his/her condition had not improved at all (nJA30). Table 2 shows the areas in which patients who participated in the survey previously perceived poor care regarding different aspects. Furthermore, it was assessed to what extent patients experienced health literacy-sensitive communication (e.g., “I was asked if I understood any information or documents” and “I was encouraged to ask questions when I didn’t get something”) according to Ernstmann et al. [14]. Here, *N* = 39 patients rated the extent of health-literacy sensitive communication on a scale from 1 (no agreement) to 4 (full agreement), with an average score of 2.97 (SD = 0.63).

#### 3.4.5. Organizational Processes/Framework Conditions

Compared to JA patients, nJA patients perceived applying for medical rehabilitation as both quick and straightforward (nJA31). The process was often initiated by the interviewees’ general practitioners (nJA32), although other health care providers, such as orthopedists, were also mentioned as initiators in some cases (nJA33). Contrary, JA patients perceived applying for medical rehabilitation as challenging (JA42). Others felt “caught in the middle” because it was unclear which funding agency was responsible (JA43). Furthermore, while nJA patients reported there were sometimes (too) many appointments, making it difficult for patients to attend all of them on time (nJA34), JA patients reported that appointments perceived as important were scheduled too infrequently (JA44). Also, the waiting time for appointments with specialists outside the rehabilitation setting was described as very long (JA45), and patients sometimes had to repeatedly advocate for their appointment requests before they were scheduled (JA46).

*“Sometimes, even if you could bend yourself, you wouldn’t make it to appointments on time.” (nJA34, RH-02)*

*“I also took care of it myself [the appointment with a psychologist], even though I had written in the flyer that I, uh, feel uncomfortable at work (…)” (JA46, RH-13)*

The effects of the SARS-CoV-2 pandemic caused significant difficulties for some patients, with emotional and practical impacts of the pandemic on the rehabilitation experience, from strict mask mandates and limited access to facilities, to the inability to receive visits from loved ones—factors that significantly affected the patient’s motivation and wellbeing (nJA35).

The questionnaire assessed to what extent patients have previously experienced “insufficient care”, showing that the majority has not perceived insufficient care so far. The results can be found in Table 3.

### 3.5. Corporate Integration Management (BEM)/Support for Participation in Working Life (LTA)

Statements regarding areas of corporate integration management and support for participation in working life were predominantly found in the JA group, where patients reported both positive and negative experiences. For example, some patients shared positive experiences, such as the provision of technical aids that facilitated normal work activities (JA47). Further, services from the integration specialist service (Integrationsfachdienst, IFD) were utilized, and BEM meetings were either offered or conducted. Established structures and support at work were experienced as helpful (JA48). However, there were also hindrances, such as situations where employees experienced fear of BEM meetings due to negative experiences shared by colleagues (JA49). Additionally, there were processes that were supposed to support the employee but were hindered by the employer, such as with BEM (JA50) or in relation to the IFD (JA51). These statements illustrate both the support that some patients received, such as technical aids and assistance from specialized services, and the barriers they faced, including fear and resistance from employers in engaging with reintegration processes.

*“Yes. Uh, in addition, I now get, because the speech computer doesn’t work, brand new touch monitors with touchscreen, so I don’t have to use the keyboard anymore. (…) This makes working easier for me. (…) Yes, I/I assume I’ll be able to work normally again.” (JA47, RH-16)*

*“Then after that, they were really angry when I got the 30 percent disability rating and brought in the Integration Office. They were totally dissatisfied, didn’t understand it at all. They had to fill out an application for the employer to get some support from the Integration Office. That still hasn’t happened. They just didn’t want to do it.” (JA51, RH-13)*

**Figure 2:**
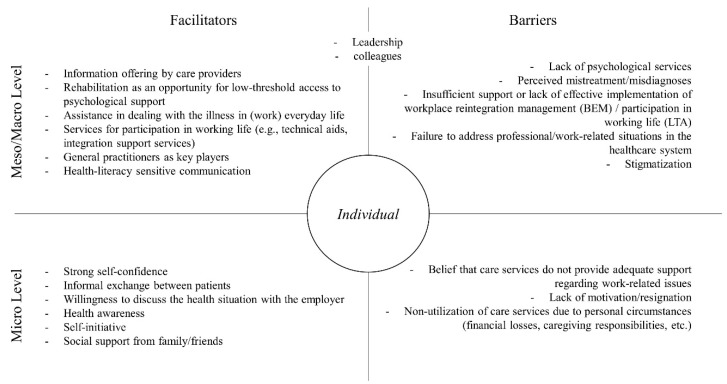
Barriers and Facilitators.

In relation to the presented results, various facilitators and barriers can be identified both at the micro level and the meso/macro level (see Figure 2). Interviews highlight the lack of psychological services, misdiagnoses, and stigmatization as key barriers to healthcare and work-place reintegration. Insufficient support for workplace reintegration management (BEM) and participation in working life (LTA) might further hinder recovery. Personal barriers such as resignation, financial challenges, or caregiving responsibilities often prevent individuals from accessing care services. Facilitators include clear information from providers, low-threshold access to psychological support during rehabilitation, and tools like technical aids for workplace participation. Supportive general practitioners, health-literacy sensitive communication, and self-initiative play crucial roles in bridging health and work challenges. Additionally, informal patient exchanges, employer understanding, and strong social support from family, friends, colleagues or leaders might foster resilience and recovery.

## 4. Discussion

The results indicate differing experiences between JA and nJA patients. nJA patients often forgo treatment due to familial obligations but benefit from strong social networks and financial stability, enabling better access to healthcare and professional opportunities. In contrast, the here investigated JA patients reported negative familial dynamics, financial barriers, and a greater need for emotional and practical support. Workplace experiences varied, with nJA patients benefiting from works councils and task adjustments but criticizing unfulfilled promises, whereas JA patients faced more barriers like employer refusals to accommodate their needs. Psychological support remains an unmet need for JA patients, while nJA patients report positive but sometimes skeptical experiences with these services. nJA patients’ occupational concerns were more frequently addressed during treatment, whereas JA patients noted a lack of focus on their work situations. JA patients often had to self-advocate for reliable health and work-related information, unlike nJA patients, who received clearer guidance. Organizationally, nJA patients found applying for rehabilitation more straightforward, while JA patients faced delays and un-clear responsibilities. The COVID-19 pandemic introduced emotional and practical difficulties for both groups.

Overall, the results of the interviews as well as the survey are very heterogeneous and reveal possible barriers and facilitators for both groups. Future studies could test the hypothesis that work-related anxiety is a moderating factor influencing the impact of various barriers and facilitators on return-to-work outcomes.

Specifically, barriers such as the lack of psychological services or employer support and mistrust of the healthcare system or the employer might prevent individuals from receiving necessary support. Yet, prompt support would be important especially for patients with work-related anxieties, as prolonged work absences can further reinforce existing work-related anxieties [16,17]. Moreover, personal barriers such as financial stress or caregiving responsibilities can also hinder the utilization of health services, as individuals often prioritize other obligations over their well-being, making it difficult to access necessary resources. These personal challenges are further compounded by the lack of trust in healthcare providers, which can discourage individuals from seeking the support they need. In a qualitative study on obstacles to and facilitators of return to work for patients with work-disabling back pain, Dionne et al. [18] found hindering aspects that are similar to the results presented here, including family aspects, occupational difficulties (e.g., lack of understanding), financial difficulties, and difficulties related to health services (e.g., lack of information).

In contrast, several facilitating factors may help improve individuals’ ability to manage health challenges and reintegrate into the workforce. Based on the results, information from healthcare providers is critical in helping individuals understand their condition and available support options. Furthermore, rehabilitation provides a chance to step back from every day (work) life, allowing individuals to focus on recovery and access psychological support in a low-threshold setting. Furthermore, support in managing health challenges in everyday work life, such as through technical aids and integration services, is essential for a successful return to work. Also, the relevance of individual factors becomes evident. Personal circumstances (e.g., social support, self-confidence, motivation and proactive behavior in seeking help), might help in overcoming barriers and accessing necessary care. Also, Dionne et al. [18] identified similar facilitators regarding return to work, including personal factors (e.g., personal attitudes and beliefs), and occupational factors (e.g., improved work conditions, gradual return to work options, employer support). However, while Dionne et al. [18] describe the need for individuals to (financially) support their families as a facilitating factor in returning to work, this study found it to be primarily a barrier to utilizing care services. The perceived necessity, from the perspective of those affected, to be present for their families can therefore be interpreted both as facilitating and hindering.

Particularly regarding work-related anxieties, which can lead to prolonged periods of sick leave in advanced stages [9], it is crucial to focus on preventive measures and involve both employers and health professionals, taking into account both the individual and system level. A potential approach here could be the concept of health literacy. Health literacy “entails people’s knowledge, motivation and competences to access, understand, appraise, and apply health in-formation in order to make judgments and take decisions in everyday life concerning healthcare, disease prevention and health promotion to maintain or improve quality of life during the life course” [19]. However, it is crucial to emphasize individuals’ skills and abilities while also accounting for the demands and complexity of the system they navigate [20]. While individual health literacy focuses on a person’s specific skills and abilities, organizational health literacy “is described as an organization-wide effort to transform organization and delivery of care and ser-vices to make it easier for people to navigate, understand, and use information and services to take care of their health” [21]. Furthermore, health literacy-sensitive communication is linked to patient enablement, with strengths identified in explaining examination results and using slow, clear language. The moderation of this relationship by physician support emphasizes the significance of patient-physician relationships and the critical role of physicians in patient care [14]. Communication also plays a crucial role in the workplace. For example, the way leaders communicate with employees influences employee satisfaction [22]. Additionally, health-supportive leadership positively impacts employee well-being [23], and leaders play a significant role in the return-to-work process [24].

The importance of mental health literacy is particularly noteworthy in the studied context. Specifically, the mental health literacy of both workers and their supervisors is a crucial factor in establishing early intervention and support for employees facing mental health challenges [25]. Finally, the concept of work-related health literacy provides another potential approach. According to Ehmann et al. [26], work-related health literacy involves employees’ understanding of their work-related risks, threats, and requirements, their own working conditions and needs, and available support both within and outside the workplace.

### Strengths and Limitations

The patient selection was quite selective, with few patients exhibiting significant health- or body-related anxieties or workplace phobia. However, we chose this approach because we also wanted to identify facilitating factors that prevent such fears from arising. In order to understand what leads to a good balance between health and work, we had to include a broad range of patients. Additionally, all statements were made from a single perspective, as the employers’ or health care providers’ viewpoint was not considered. The interviewees may, for example, suspect bad intentions from the employer behind the workplace reintegration (BEM) discussion, while the employer may actually have good intentions. This could be an interesting area for further research. It is important to note that the highlighted differences between JA and nJA patients may serve as a basis for further research, but they do not allow for generalizable conclusions and the same applies to the quantitative data, which are based on a convenience sample of heterogeneous origin and therefore do not offer a reliable description of the workforce.

Given the broad scope of the research question and limited resources, the possibility of continuing interviews until theoretical saturation was reached was constrained. However, as noted by Akremi [27], crucial themes can still be identified even with smaller sample sizes. Furthermore, there may be a tendency for differences in the quantitative data between patients in medical rehabilitation and those in the orthopedic clinic. However, these differences were not statistically tested due to small sample size and require further investigation. Instead, the quantitative data were integrated with the qualitative findings in order to support the insights drawn from the qualitative analysis of the barriers and facilitators to work recovery. This may limit readers’ ability to independently verify the quantitative findings.

## 5. Conclusions

Untreated work-related anxiety can contribute to prolonged work disability, increased healthcare utilization, and substantial economic costs. Our findings illustrate significant differences between JA and nJA patients in terms of their experiences, challenges, and support needs within healthcare, workplace, and rehabilitation contexts. JA patients face more obstacles and therefore require greater support compared to nJA patients, e.g., improved employer accommodations, accessible psychological support, and enhanced communication of health-related information. Patients with job anxiety realized that they are in need of targeted work-related interventions and feel these come somehow too short. From a public health perspective, the identified barriers suggest possible gaps in care pathways that, if confirmed in larger studies, could be addressed through early, integrated screening upon rehabilitation admission and targeted interventions specifically designed to meet the needs of patients with work-related anxiety, with the goal of improving return-to-work outcomes and reducing work-related disability.

While the findings provide qualitatively valuable insights, they remain pilot and explorative rather than generalizable and call for further research to explore these patterns and their implications in broader populations. Also, the associations between the various factors and their impact on the (non-)utilization of care services and/or professional support remain to be clarified.

## Figures and Tables

**Table 1:** Professional relevance in the care provision.

	N (Rehabilitation)			N (Orthopedic Clinic)		
	(somewhat) agree	(somewhat) disagree	I cannot assess this.	(somewhat) agree	(somewhat) disagree	I cannot assess this.
During my stay at the rehabilitation clinic/orthopedic clinic, my personal professional situation was sufficiently taken into account.	18	14	2	8	5	3
During my stay at the rehabilitation clinic/orthopedic clinic, my needs regarding my professional situation were adequately addressed.	18	12	3	10	3	3

**Table 2:** Perceived poor care.

	N (Rehabilitation)			N (Orthopedic Clinic)		
	Yes, at least once.	No.	I don’t know.	Yes, at least once.	No.	I don’t know.
I have previously received poor care because the responsible healthcare providers did not communicate or coordinate well with each other.	12	16	2	9	7	1
I have previously received poor care because the responsible healthcare provider did not properly review my medical file.	9	16	6	6	9	2
I have previously had negative experiences with doctors or other healthcare providers who did not take my personal needs seriously.	21	10	1	8	7	2
I have previously had negative experiences with doctors or other healthcare providers who did not take my health problems seriously.	19	12	1	10	7	0
I have previously had negative experiences with doctors or other healthcare providers who did not take my professional problems seriously.	14	12	4	7	7	3
I have previously been unable to access healthcare services because deadlines or waiting times were not met.	6	21	4	5	10	2

**Table 3:** Perceived insufficient care.

	N (Rehabilitation)			N (Orthopedic Clinic)		
	Yes, at least once.	No.	I don’t know.	Yes, at least once.	No.	I don’t know.
I have previously not received healthcare services (e.g., physiotherapy) because my doctor no longer had any available quota for it.	11	15	4	8	5	4
I have previously not received healthcare services because I could not get an appointment with the responsible specialist.	13	17	1	8	6	3
I have previously not received medical care because it was unclear who was responsible for me.	5	24	2	6	10	1
I have previously not received professional assistance because it was unclear who was responsible for me.	4	21	4	3	7	7

## Data Availability

Due to German data protection standards, interview data cannot be made publicly available. Anonymized survey data are available from the corresponding author on reasonable request.

## Supplementary Materials

The following supporting information can be downloaded at: https://www.mdpi.com/article/10.3390/ijerph23010125/s1, Table S1: quotes.

## Abbreviations

The following abbreviations are used in this manuscript:

JA	Higher Job Anxiety Scale scores
nJA	Lower Job Anxiety Scale scores
