# Supplementary material for: Opportunities and Challenges in the Care of Patients with Somatic Complaints and Patients with Additional Work-Related Anxiety—A Mixed Methods Study
Source: Int J Environ Res Public Health. 2026 Jan 20;23(1):125. doi: 10.3390/ijerph23010125 (PMC12840998; doi:10.3390/ijerph23010125)
Supplement: Supplementary file 1 [file ijerph-23-00125-s001.zip › ijerph-3999040-supplementary.pdf]

Supplementary Table S1: quotes

Individual situation					
Social environment					
Ref.	Quote	patient	Ref.	Quote	patient
JA1	"Sure, I talked a lot about it with my friends and also with my mom."	RH-04	nJA1	"Last time, my husband was able to visit me here. (…) Uh, that really helps, you know? It lifts you up a bit, gives you a little boost, you know? Like, 'Come on now, push through this.'"	RH-08
JA2	"Uh, during the time when I only had my back problems, of course, they would say, 'Stop lifting heavy things.' And WHEN I did try to lift something heavy, I'd immediately get scolded for it. Uh, my mom usually sets things up for me in a way that I don't have to bend down."	RH-04	nJA2	"And my wife works in the hospital, and she said, 'That's it, enough is enough,' packed my bag, and off we went."	RH-15
JA3	"She [sister] said, 'Let me check,' and she looked on the [Pension Insurer] website. Ah, rehab, your age, illness – she wrote everything down."	RH-07	nJA3	"(…) I have two sons. The older one had already moved out, and with the younger one, who was 6 years old, I was then a single parent and simply couldn't work shift work anymore."	RH-08
JA4	"(…) since my [family member] was the boss in the (…) department there and he has a bit of a strange character (laughs), you know, a bit of a "know-it-all" and a bit of a control freak. (…) they asked, 'Are you related to [Name]?' I said, 'Yes, I'm his [family member].' That was it. They started ignoring me and... well."	RH-06	nJA4	"It was supposed to be a fifth week, uh, but that would have been over Christmas, (…) and since I'm rarely at home, of course, I have to travel. Uh, I have six grandchildren, and I can't keep putting them through it with Grandpa constantly being away."	RH-03
JA5	"But, uh, my [family member] passed away when I was [age], and then my [family member] came into the picture. And then things weren't so nice anymore. (crying) As a result, I had to start working at home from a very young age, running the household, looking after my siblings later on. Yeah. And like I said, all of this probably contributed to the fact that I couldn't physically handle it, given my body type."	RH-06	nJA5	"(…) I also got an appointment a bit quicker through, let's say, a family member of a friend, who is an orthopedist – since it's hard to get appointments, I managed to get one faster."	RH-08
JA6	"My father was also like that – a work machine, a workaholic, just like me. I'm like him. (laughs)"	RH-07			
JA7	I: "And, uh, through whom would you have wanted more support during all this time?" P: "Through whom? (exhales loudly) Family. The family doesn't say anything. Quiet. They could also help me a bit. My wife hasn't worked a single day, not one day. She's a housewife. Well, she had serious illnesses ten years ago, breast cancer and such, so I've always been calm. I work, I provide for everything. Uh, pff, yeah, maybe	RH-07			

	friends could have said something here and there. No one said anything, no one wants to say anything. I don't know."				
JA8	"I know a few people, a couple of friends, who had surgery and now can't walk anymore. That's why I'm afraid. I don't want to do it."	RH-07			
Financial situation					
Ref.	Quote	patient	Ref.	Quote	patient
JA9	"And now I have to buy a new mattress. I say, 'Yes, we don't have the money right now, just wait a bit (…)."	RH-07	nJA6	Yes, privately. I paid for it [outpatient rehabilitation] myself."	RH-14
JA10	"I would like to stay for another week, but if the timing doesn't work out and I don't get any money, then I'll have to go home, with the Hamburg model, and just manage."	RH-05	nJA7	"Uh, my children are both adults now, standing on their own feet, so I'm no longer under the pressure of needing to keep the job to support the family. Uh, that pressure is thankfully gone, and I don't worry about it now because either it works, or it doesn't. If not, I'll just have to find something else."	RH-02
Attitudes and beliefs					
Ref.	Quote	patient	Ref.	Quote	patient
JA11	"If my HR manager knows I have back problems (laughs), I don't think he really cares about that anyway."	RH-10	nJA8	"Well, how is he supposed to react? There are no options to react."	RH-03
JA12	"And since, unfortunately, I'm the type who can be told anything, uh, I said I wouldn't attend this meeting, such a conversation. For my own safety. I didn't say that, but – "	RH-05	nJA9	"Actually, the focus was more on physical intensity, that I/ that I first get fit again. You can't address every profession, uh, for example, a chef or a carpenter, or/ that's just not feasible. Because you're usually in a group except during physiotherapy, but I couldn't even imagine how that would work. There would have to be another 20 or 30 therapists running around, tailored to every professional field. And if I haven't worked on that myself yet, I wouldn't even have the experience, so it's just not doable."	RH-15
JA13	"Partially, of course, also with a certain pressure, which I believe every boss or every large corporation applies."	RH-04			
JA14	"Nobody is satisfied with their workplace, no one."	RH-06			
JA15	"(…), something has to change, I can't always be carrying heavy things. And, uh, that they should come up with something as well."	RH-04			
JA16	"I can't work. I want to work, but I CAN'T. I'm done; this has really worn me out. Well, even if I do three weeks of rehab here now, I don't think I'll be fit again with my spine."	RH-07			

(planned) behavior					
Ref.	Quote	patient	Ref.	Quote	patient
JA17	"Uh, I actually need/ well, I have an appointment with the social worker because I want to ask about the possibility of retraining."	RH-04	nJA10	"uh rather, uh, to look for something new, yes, maybe through the pension insurance, uh, either to do retraining or, uh, maybe (…)"	RH-12
JA18	And at first, I had very few absences because I never let myself go and always got up and went, even when I couldn't walk."	RH-16	nJA11	"I don't go to the doctor for every little thing, you know, that's just how it is. I also go to work (laughing) even when I'm in pain. That's just how it is, you know. I take an ibuprofen or even two, and then I get going."	RH-08
JA19	"And somehow, I'm lacking the strength or something, that initial moment where I say, fine, then I'll quit here and look for another job."	RH-13	nJA12	"You know it, but you still don't do it."	RH-17
			nJA13	"Of course, I won't be doing that in [Place5] anymore. I can't walk around in [Place5] with a Nordic walking stick. People would just shake their heads and say I'm crazy. But here, I stick with it, and it does me good."	RH-09
			nJA14	"You could definitely try at work (takes a deep breath) to get a different position – I don't know. I definitely want to try for something calmer." RH-09-26-09, and "well, I think being on the ward now/ no, that wouldn't be good for me. I've already organized everything for myself. I realized it; I didn't even have my 50 kilos here anymore, I was so thin, and I knew it couldn't go on like that. So, I've taken care of myself, yes."	RH-18
Wishes about work and health					
Ref.	Quote	patient	Ref.	Quote	patient
JA20	"Mhm (thinking), it would be important for employers to actually do more. Do more for the health of their employees, not just in terms of workload itself, but also regarding workplace safety and everything else. (...) I think there needs to be a strong focus on ensuring that employees truly feel well taken care of and enjoy going to work, because honestly, I think that's something very few companies provide nowadays, especially large companies. At the moment, I have to say, I don't know anyone who says, 'I'm excited about going to work."	RH-06	nJA15	"That they simply listen sometimes, that the people who have something to say maybe just join us for a few shifts. (laughs) Yeah, because I always had the feeling that they think we're exaggerating."	RH-18
JA21	"(…) I don't know, maybe you spend half a day at the register and half a day outside. Just to have some variety and not just this one-sided monotony."	RH-04	nJA16	"What I would really appreciate is if they provided us with a room at work with some exercise equipment. The opportunity is definitely there. We actually have the time for it; we have a	RH-08

				LOT of downtime, a lot of standby time, where we could go there, take a radio with us, and say, 'Hey, I'm here, let me know if you need me."	
JA22	"I just wish for someone who doesn't call me into the office every time and accusingly put an illness chart in front of me."	RH-13	nJA17	I: "What kind of appointments should those be? I think you're referring to those group sessions you mentioned." P: "Yes, those could be dropped, and more focus placed specifically on me. I have this great course in physiotherapy—it's all about me. I do my exercises, and the therapist focuses only on me. She tells me exactly, 'Move your shoulder back, lift your pelvis,' and she monitors it at the same time to make sure I'm doing it right for when I'm at home. That's the kind of thing I want more of."	RH-11
JA23	"So, I would wish that people who get sick receive a better flow of information on their own, without having to figure everything out themselves. They really have to work for EVERYTHING (…)"	RH-16			

**work**					
Company support / offers					
Ref.	Quote	patient	Ref.	Quote	patient
JA24	"(…) at [employer], we have a special workplace team, the representative body for severely disabled employees, the integration specialist service, and the staff council, and I am well supported there."	RH-16	nJA18	"We actually had a really great works council. He would even come to our ward and see things for himself. So, he was really engaged with us, I'd say. He was also a caregiver for a long time and still is, you know, and he even worked on the ward. He really tried to make a difference, yeah."	RH-18
JA25	"They also had to fill out an application so that the employer could get some support from the integration office. That still hasn't happened to this day. They simply didn't want to do it."	RH-13	nJA19	"Due to the illness, it eventually got to the point where, as I said, I just couldn't do it anymore. But they know that, so they're already trying to take me out of some tasks a bit. I'd rather drive the car than do other things."	RH-09
JA26	"Accordingly, we've already had many discussions about this, and (…) to date, they are not willing (exhales audibly) to actually provide an alternative workplace."	RH-06	nJA20	"We felt completely abandoned. We were so often told that we would get relief through more staff, that renovations would be made because the layout wasn't optimal, and so on and so forth. No, I mean, for years we were put off with promises that things would get better, that we'd always have three people on night duty, and so on. No, I've given up believing that. We turned to the works council back then and so on. Oh, no, I've	RH-18

				long since lost faith in that. Yeah, I have. You can forget about it."	
Leadership and colleagues					
Ref.	Quote	patient	Ref.	Quote	patient
JA27	"(…) but then I had a new boss, and she was very supportive. She asked, 'What are your strengths?'"	RH-16	nJA21	"He was very understanding, and uh, I had no issues."	RH-14
JA28	"(…) my bosses don't want to find any options for me anymore."	RH-16	nJA22	"(…) and uh, he also tries to support me a bit because I said I can't do it anymore."	RH-09
JA29	"(…) she removes me from my group. I had a fixed group, and it worked really well, and uh, no one was up-set—not the parents, not the colleagues—only my boss, because I kept having to leave for doctor's appointments."	RH-13	nJA23	I: "Uh, you mentioned earlier that you can imagine people talking behind your back." P: "Mhm (Affirmative)." I: "At what level, like who, who do you think it is?" P: "(...) The boss himself too."	RH-02
JA30	"(…) so, there was a restructuring, and we were all reassigned. I just asked if I could sit near the heater. I didn't ask for anything more. And then my boss said to me, 'We're not a disability department here.'"	RH-16	nJA24	"So, uh, you could say/ no, last year I had a car accident (...) and then, for example, a colleague saw that it was my car and told the boss. The first thing my boss said, for example, was: 'Oh, I'll have to replace [Name].'"	RH-12
JA31	"Then I was a bit sick, and my boss kept telling me, 'Oh, [Name], you were very good, you were good, good, and now sick, sick, that's not possible.' And then he fired me."	RH-07	nJA25	"(…) she only works early shifts, from morning until a maximum of 10 a.m., because she has to go to dialysis after that. You can be grateful that we can still keep her on, that she at least has some distraction and is supported in her work. She also has a small child, which makes it bad enough that she has this illness. And then some colleagues complain, saying, 'She only works early shifts,' and I just want to slap my forehead."	RH-03
JA32	"(…) if I didn't have these people, I would have already, uh, gone under."	RH-16			
JA33	"(…) yes, they always say, of course, that it's obviously unfortunate, uh, but not because I wasn't there, but rather because I'm still so young and, um, that there really shouldn't be any health problems at such a young age."	RH-04			
JA34	"Well, everyone wanted to be better. They did tasks that, just a week earlier, they would have said, 'Are you crazy, carrying that on your own?' and so on. You can't imagine it – they pushed themselves to the limit, just to show, 'I'm not going to be laid off.' It was like something out of a book or a bad movie. (...) A week earlier, we were all still having nice breakfasts together, and the following week, mm-hmm, they skipped their breaks. (laughs)"	RH-05			

Healthcare					
Psychological/psychotherapeutic services					
Ref.	Quote	patient	Ref.	Quote	patient
JA35	"I told the doctor that I... I included everything in the application. Contact with a psychologist, yes. I think if you talk to someone and they give you advice, it might make a difference."	RH-05	nJA26	"So, it's not really bad, this, let's say, whole psychology stuff [group sessions], but it's, uh, yeah—it does help, I think. As long as it's not too much or anything. I don't know, but if you have two or three appointments during those three weeks, then that's fine, yeah (affirming)"	RH-17
JA36	"I said I would like a psychological session. I also said it here and did NOT receive one."	RH-16			
Addressing work situation during treatment phase					
Ref.	Quote	patient	Ref.	Quote	patient
JA37	I: "Okay. And to what extent do these doctors address your work situation?" P: "Not at all. They're not interested. Not at all. Zero." I: "Would you have wanted that?" I: "Uh, yes. (laughs) Yes. (…)" or I: "So you're saying your work situation was only brought up during the initial consultation and then not at all?" P: "Not even then. It was only asked: 'Do you want to retire?' I said no. That was it. (…) There were no further questions, nothing. I was completely surprised, but no follow-up at all. I found that very unusual."	RH-16	nJA27	"Yes, I mentioned the psychologist and the rehab counselor—that was very, very helpful. You have to say it like that, right?"	RH-12
			nJA28	"We talk about everything. He [the general practitioner] also asks about work processes and so on—he's very knowledgeable. We try to change some things, (...)"	RH-09
			nJA29	"I went to my general practitioner, orthopedist, and sports doctor, but none of them could really tell me what I'm allowed to do and what I'm not. Then I asked my general practitioner: (…) would I qualify for rehabilita-tion so I can finally understand how to behave? I absolutely don't want to give up my sport or my job, but someone has to tell me what I can still do and what I can't."	RH-18
Information offer					
Ref.	Quote	patient	Ref.	Quote	patient
JA38	"No, no, no. The doctor didn't do anything—he just wanted to put me on sick leave and prescribe medication. He was terrible, didn't explain anything to me (...). So I took the initiative myself, asked around, called the [self-help organization], got	RH-16			

	informational materials and brochures. I became very, very active.”				
JA39	“No one says anything. No, no one. It’s a bit different now. It used to be fine, but now you can forget it—there’s no proper information from anyone.”	RH-07			
Perceived mistreatment/misdiagnosis					
Ref.	Quote	patient	Ref.	Quote	patient
JA40	“And then he wanted to push me into the depressive category because I also have depression, but I’ve got that under control, including with medication. And he said to me, ‘Yes, back pain can also be caused by depression, so you’re in the wrong place here.’ I just thought to myself: He has my reports, my MRI reports, my orthopedic reports—I brought all the CDs. If he had looked at them before the consultation, he would have known that it’s entirely physical. That kind of thing drives me crazy.”	RH-10	nJA30	“But I was discharged with a very good prognosis, which wasn’t true. I read everything the doctor wrote. I said, ‘How am I doing? I still have the same issues.’ He said, ‘No, you’re fine.’ I said, ‘If that were the case, I wouldn’t be sitting here. I’d be back at work if I were doing that well.’”	RH-03
JA41	“I’m the kind of person who likes to get second and third opinions, just to see how others perceive the situation. One doctor didn’t even address my back at all. He just said, ‘Oh, it’s not serious—it’ll get better. Just take some painkillers and rest a bit, and it’ll be fine.’ I find it difficult when someone dismisses you like that, especially when you’ve been dealing with back pain since—what, [year] or [year]? It’s always the same issue, my discs keep slipping. I don’t want to provoke it, but I also don’t want to face the same problem every year for the next few years.”	RH-04			
Organizational processes / framework conditions					
Ref.	Quote	patient	Ref.	Quote	patient
JA42	“But it wasn’t so easy, uh, to figure everything out, and they are very hard to, uh, fill out, I think. It also says it takes 20 minutes. I was still not done after an hour and a half.”	RH-13	nJA31	“Uh, just call the pension office, and they’ll send you the forms. You fill them out and then, uh, send them back. So, it’s very straightforward.”	RH-17
JA43	“Well, I don’t know, it’s like the health insurance says they don’t want to pay sick leave anymore. And the pension insurance doesn’t want to pay a pension yet, so you’re just caught between all the chairs.”	RH-10	nJA32	“Mhm (thinking), my GP. Because back then, he said: What, you’ve never done a rehab?”	RH-09
JA44	“My first physical therapy session, which is very important, I got, um, last/ last week. So if I hadn’t gotten this extension, I would have had only two physical therapy sessions.”	RH-13	nJA33	“In consultation with my orthopedist, uh, who said it might be a good idea.”	RH-14

JA45	"It's uh/ uh of course unfortunate that you have to wait forever for specialists (...)"	RH-10	nJA34	"Sometimes, even if you could bend yourself, you wouldn't make it to appointments on time."	RH-02
JA46	"I also took care of it myself [the appointment with a psychologist], even though I had written in the flyer that I, uh, feel uncomfortable at work (…)"	RH-13	nJA35	"This whole madness with Corona here, right? It's just—doesn't work for me at all to walk around the WHOLE DAY with this mask on my face, doing sports with a mask. It just doesn't work for me. And then, as I said, you know, when I'm dealing with doctors or therapists who are supposed to help me, do something good for me, and I can only ever see them with masks—that's so depressing for me, you know? I can't have visitors here. Last time, my husband could visit me here; we had a guest room. That really helped, you know? It lifted me up a bit, gave me some energy to keep going, you know, like, 'Come on, push through this.' But now that's not possible anymore. The sauna is closed. I can't change in the swimming hall anymore. I have to walk wet, in my wet clothes and bathrobe, through the whole building after the therapy pool session and can only change and shower in my room. The training room upstairs, with all the equipment for abs, legs, arms, etc., I used to be able to use it without any problems after hours. I used that room every evening, and it really did me good, being able to use the machines there. I could use the swimming pool in the evenings whenever I wanted. None of that is possible anymore. Either it's completely unavailable, or it's only accessible with strict appointments. It's just not the same anymore, no, no. I even brought this up today during the chief physician's rounds when the topic of an extension came up. I said, 'No, not this time.' I said, 'This time, I'll do the three weeks here and call it good.' I also explained why, and surprisingly, I was met with understanding."	RH-08

Corporate integration management (BEM) / support for participation in working life (LTA)					
Ref.	Quote	patient	Ref.	Quote	patient
JA47	"Yes. Uh, in addition, I now get, because the speech computer doesn't work, brand new touch monitors with touchscreen, so I don't have to use the keyboard anymore. (...) This makes working easier for me. (...) Yes, I/ I assume I'll be able to work normally again."	RH-16			

JA48	"But I'm lucky that I'm well supported already. We have a special team for workplaces for disabled employees, the disability representative, the IFD, the works council, and I'm well supported there."	RH-16			
JA49	"Uh, we really wanted to go, but then I heard from a colleague, or she told me I shouldn't show up because, uh/ what was I going to say? There are two colleagues, the first one, who had been there for 27 years, she was also sick, came back, was called to a meeting, and, yeah, she was fired. I don't know what they offered her or anything/ because I can't imagine it. No one could be that dumb today. Fired after 27 years. And the other one, it was the same, but she hadn't been there as long. She had only been there for 12 years. Anyway, they told me not to go, they said they would pressure you. And since I'm the type who can be told anything, uh, I said I wasn't going to attend that meeting, such a conversation. For my own safety. I didn't say it, but..."	RH-05			
JA50	"Of course, we've had many conversations already, and uh... up to now, they're not willing (exhales loudly) to actually provide another workplace."	RH-06			
JA51	"Then after that, they were really angry when I got the 30 percent disability rating and brought in the Integration Office. They were totally dissatisfied, didn't understand it at all. They had to fill out an application for the employer to get some support from the Integration Office. That still hasn't happened. They just didn't want to do it."	RH-13			
